# Comparison of Speech Defects in Different Types of Malocclusion

**DOI:** 10.7759/cureus.62290

**Published:** 2024-06-13

**Authors:** Sai Vyshnavi Palakolanu, Kiran Kumar Dodda, Sri Harsha Yelchuru, Jyothsna Kurapati

**Affiliations:** 1 General Dentistry, Drs. Sudha & Nageswara Rao Siddhartha Institute of Dental Sciences, Vijayawada, IND; 2 Orthodontics and Dentofacial Orthopaedics, Drs. Sudha & Nageswara Rao Siddhartha Institute of Dental Sciences, Vijayawada, IND; 3 Orthodontics and Dentofacial Orthopaedics, Dr. YSR University of Health Sciences, Vijayawada, IND

**Keywords:** speech distortion, linguo alveolar sounds, angles classification, malocclusion, speech defects

## Abstract

Introduction

Speech has a great impact on human evolution, allowing for the widespread knowledge and advancement of tools. Difficulty in pronouncing one or more sounds is the most common speech impairment. Speech defects are more commonly associated with class III malocclusion patients (difficulty in pronouncing ‘s’ and ‘t’ sounds), the second in line is class II malocclusion (difficulty in pronouncing ‘s’ and ‘z’ sounds), and speech distortions are least affected in class I malocclusion (difficulty in pronouncing ‘s’ and ‘Sh’). Most patients with dentofacial disharmonies and speech distortions need orthodontic care and orthognathic surgery to resolve their issues with mastication, aesthetics, and speech.

Aims and objectives

To compare and assess speech difficulties in different types of malocclusion.

Materials and methods

The study was conducted over 160 subjects for three and half months. All of them were evaluated for speech defects before they received orthodontic treatment. The main basis of this study is according to Angle's classification of malocclusion. The subjects were segregated according to Angle's classification of malocclusion. Malocclusion traits that are included in this study are Angle's class I, Angle's class II division I and division II, and Angle's class III.

Results

According to the results, out of 160 subjects, labio-dental speech defects are observed in 8% where n=13 of the study participants, linguodental speech defects are observed in 2% where n=3, lingua-alveolar speech defects are present in 54% where n=86, and bilabial speech defects are observed in 2% where n=3 of the study participants. Here 'n' represents the frequency of the subjects. Severe speech defects are seen in Angle's class III malocclusion. Results according to the type of malocclusion include: labio-dental speech defects are seen in 37.5% in class I, 25% in class II division I, 0% in class II division II, and 37.5% in class III. Linguodental speech defects are seen in class III malocclusion subjects only. Lingua-alveolar sounds are seen in 27.8% of class I, 29.6% of class II division I, 1.9% of class II division II, and 40.7% of class III. Bilabial speech defects are only seen in class II division I subjects. According to the results, only lingua-alveolar speech defects are statistically significant, and more severe speech defects were observed in class III malocclusion.

Conclusion

Speech plays an important role in affecting the quality of life of people. Different types of malocclusion traits are associated with different types of speech defects.

## Introduction

Speech is an important factor in every individual’s daily life. Speech is a complex process that is associated with various other processes like respiration, phonation, resonation, articulation, etc. Air that is released from the lungs travels along the trachea and vocal cords in order to produce various sounds, which include high-pitch and low-pitch sounds. The tongue is the most important factor to consider for helping in speech production [1].

The tongue, which is positioned within its structural boundaries, namely, the dentition and dental arches, plays an important role in the articulation and production of sounds [2]. Most of the consonants are produced due to the interaction of the tongue with the anterior oral cavity. Deviation in the alignment or defective dental arches may result in abnormalities of speech articulation [3].

Various abnormalities in speech production have a negative impact on the lives of people [4]. Most of the people are affected psychologically due to speech production abnormalities [5]. The majority of the patients develop an inferiority complex that negatively impacts the psychological health of the patient. People with severe speech defects may not be able to develop normal communication skills and develop psychosocial disorders [4].

Speech defects can be easily identified and can be corrected by early orthodontic treatment. Orthodontic treatment is not only limited to the correction of malocclusion of the teeth or restoration of their function but also focuses on the restoration of speech defects in the patients, which helps them lead a quality life.

Speech sound disorders were previously studied for various etiological factors and it was found that they are heterogeneous and can be idiopathic, genetic, or acquired in nature. Common causes of speech defects include malocclusion, cleft lip, cleft palate, ankyloglossia, and so on. The most common occlusal anomalies that have a negative impact on sound production are open bite, mandibular prognathism, and retrognathism.

Anterior open bite can result in abnormal production of interdental fricatives and a class III reverse overjet can affect the articulation of labiodental sounds like f and v [6]. Variations in the production of vowel sounds were observed in class II malocclusions due to the adaptive changes in tongue placement [7]. Although previous studies have shown that occlusal anomalies and tongue position are the causative factors for speech disorders, language may also play an important role. As literature is scarce regarding the association of tongue and malocclusions in speech defects, the current study was proposed.

The current study focused on determining the effect of different malocclusions on the production of sound using auditory and visual descriptions. The hypothesis was that the type of malocclusion does not affect the sound production.

## Materials and methods

After receiving approval from the Institutional Ethical Committee, Drs. Sudha and Nageswara Rao Siddhartha Institute of Dental Sciences (Ref. no: IEC/DRS.S&NRSIDS/2024/PG/35), outpatients visiting the Department of Orthodontics and Dentofacial Orthopaedics for orthodontic treatment were selected for the study and informed consent was taken. The study spanned over a three-and-a-half month period and a total of 160 patients seeking orthodontic treatment were included.

All the subjects meeting Angle’s classification of malocclusion (class I, class II division 1, class II division 2, and class III) who did not receive any orthodontic treatment were included. Patients presenting with a cleft lip and palate and with neurological and mental disorders were excluded from this study.

The study pool comprised 69 male and 91 female patients between the age groups of 9 and 35 years.

Table 1 represents the distribution of subjects according to their malocclusion.

**Table 1:** Distribution of types of malocclusion in the given subjects

Type of malocclusion	No. of subjects
Angle's class I	40
Angle's class II division 1	40
Angle's class II division 2	40
Angle's class III	40

A minimum age of nine years was considered because the oral motor structure and normal speech sound production are considered mature and well-integrated by this age. Subjects with anatomic and physiologic anomalies like mental retardation, neurological disorders, cleft lip and palate, ankyloglossia, presence of chronic habits, previously diagnosed speech disorders and hearing difficulties, and previously orthodontically treated patients were excluded.

All the subjects who participated in the study speak Telugu in addition to English as the primary language at home. This shouldn’t be considered an exclusion criterion as there are not many linguistic differences between Telugu and English pronunciation.

After obtaining consent from the study sample, a detailed evaluation was done by the primary investigator who assessed the subject for the following variables: 1) overjet, 2) overbite, 3) Angle’s malocclusion, 4) crowding, 5) spacing, etc. Each subject was evaluated for labiodental, lingua-alveolar, linguodental, and bilabial speech defects with imitation of verbal sounds, and their traits of malocclusions were identified according to Angle’s classification.

A total of 160 patients were selected and divided into 4 groups of 40 each, according to Angle’s classification as Angle’s class I with crowding and spacing, Angle’s class II division 1, Angle’s class division 2, and Angle’s class III malocclusions. In all the groups, speech defects were evaluated as given below.

Subjects are asked to pronounce some consonants. For the evaluation of labiodental sounds, the subject was asked to count from “fifty-one” to “fifty-nine”. For the evaluation of lingua-alveolar sounds, subjects were asked to pronounce “sixty-one” to “sixty-nine”. The evaluation of linguadental sounds was made by asking the subject to pronounce words like “this”, “that”, “these”, and “those”. Careful observation of these words provides the evaluation of linguodental sounds. And the evaluation of bilabial sounds was made by asking the subject to pronounce letters like “B”, “P”, and “M”.

## Results

According to the results of 160 subjects, labiodental speech defects are observed in 8% (n=13) of the study participants, linguadental speech defects are observed in 2% (n=3) lingua-alveolar speech defects are present in 54% (n=86), and bilabial speech defects are observed in 2% (n=3) of the study participants. Here, n = frequency (number of participants affected with speech defects).

Table 2 represents the frequency distribution of speech defects in the overall study participants.

**Table 2:** Different types of speech defects present or absent in the given subjects along with their percentages

S.No	Speech defect	Present or absent	Percentage %
1.	Labiodental	Present	8
		Absent	92
2.	Linguodental	Present	2
		Absent	98
3.	Lingua-alveolar	Present	54
		Absent	46
4.	Bilabial	Present	2
		Absent	98

The collected data are entered into Microsoft Excel (Microsoft Corporation, Redmond, WA, USA) and subjected to statistical analysis using SPSS version 21.0 (IBM Corp., Armonk, NY, USA). Frequencies were calculated for each group, followed by a chi-square test to analyze categorical variables. The statistical significance level was set as p<0.05*.

Table 3 represents the distribution of speech defects in relation to the malocclusion trait.

**Table 3:** Type of speech defect and percentage of speech defect present or absent in the given subjects Chi-square test, Statistical significance set as p<0.05*. The value of the data <0.05 is considered as statistically significant. So in the given data only linguo-alveolar speech defects are statistically significant because they have the p value <0.001.

Speech defect	Present or absent	Class I	Class II Division I	Class II Division II	Class III	P-value
Labio-dental defect	Present	37.5%	25.0%	0	37.5%	
	Absent	34.8%	32.6%	5.40%	27.2%	p<0.834
Linguodental defect	Present	0	0	0	100%	p<0.158
	Absent	35.7%	32.7%	5.1%	26.5%	
Lingua-alveolar defect	Present	27.8%	29.6%	1.9%	40.7%	p<0.001**
	Absent	43.5%	34.8%	8.70%	13.0%	
Bilabial defect	Present	0	100	0	0	p<0.220
	Absent	35.7%	30.6%	5.10%	28.6%	

According to the chi-square test, lingua-alveolar sounds are statistically significant. Among the study participants, most of them have difficulty pronouncing lingua-alveolar sounds. Also, the subjects with class III malocclusion have shown a higher incidence of speech difficulty in pronouncing lingua-alveolar sounds followed by class II malocclusion, and subjects with class I malocclusion have shown the least incidence of speech defects in lingua-alveolar sound pronunciation.

## Discussion

The production of speech defects negatively impacts people’s ability to lead a quality life and harms their quality of education and educational outcomes. Most people come to the Department of Orthodontics and Dentofacial Orthopaedics for aesthetics and smile corrections [8]. Orthodontic treatment mainly focuses on resolving aesthetic, speech, and masticatory problems. However, aesthetics and pronunciation are important functions of anterior teeth. Pronunciation and aesthetics are influenced by many factors, in which malocclusion is the most important and common factor to consider in most people. Determining the possibility and cause of speech defects will help to resolve any speech-related issues. When defining aesthetics, most commonly, pronunciation is overlooked [9]. But, pronunciation (speech production) is an important factor that we need to consider. Speech production mainly depends on the position of anterior teeth and the position of the jaw [10]. Speech defects and dental malocclusions may have various etiological factors that may also be due to various genetic factors [11]. Various other factors should also be taken into consideration when determining a speech defect, as many other reasons may contribute to speech production, namely, the position of lips, whether the lips are competent or incompetent, the position of the tongue, and the position of anterior teeth [12]. The present study was conducted over 3.5 months, in which we focussed on comparing different types of speech defects in different types of malocclusion. As we know, there are many traits of malocclusions, namely, Angle’s class I, class II division 1, class II division 2, class III, open bite, crossbite, crowding, spacing, and so on. Angle's classification and their molar relationship are as follows: In Angle's class I malocclusion, the mesiobuccal cusp of the maxillary first molar comes in contact with the buccal groove of the mandibular first molar. In Angle's class II malocclusion, the distobuccal cusp of the maxillary first molar comes in contact with the buccal groove of the mandibular first molar. In Angle's class III malocclusion, the mesiobuccal cusp of the maxillary first molar comes in contact with the buccal cusps of both the mandibular first and second molars [13]. Various types of sounds are listed as labiodental or fricative sounds, lingua-alveolar sounds, linguodental sounds, and bilabial sounds. In labiodental or fricative sounds, the incisal edges of upper anterior teeth touch the lower lip line (‘F’ or ‘v’). In lingua-alveolar sounds, the tip of the tongue touches the palatal rugae (‘s’ or ‘Ch’ or ‘j’) also known as sibilant sounds [14]. In linguodental sounds, the tip of the tongue lies in between the upper and lower teeth (‘this’ or ‘that’) while pronouncing bilabial sounds, both the upper and lower lip will be in contact (‘B’ or ‘M’). The study was conducted with over 160 subjects who came for consultation at the Department of Orthodontics and Dentofacial Orthopaedics and agreed to participate in this study. All the subjects were evaluated before they received orthodontic treatment. Out of them, 40 had Angle’s class I malocclusion, 80 subjects had class II malocclusion (40 of them with class II division I, and the other 40 subjects with class II division II), and 40 subjects had class III malocclusion and were evaluated for the production of sounds, namely, /s/, /t/, /b/, /p/, /m/, etc. It can be assumed that when the diagnosis is more specific, the interpretation will be even more accurate and more specific as well. In this study, most of the subjects presented with Angle's class II malocclusion followed by class I and class III. The results of this study are similar to other studies [4] in which the subjects with class III malocclusion have the highest incidence of speech defects. There are certain limitations in this study like the number of subjects; as it is a short study, we included only 160 subjects. The other limitation is types of malocclusion, which are only limited to Angle's class I, class II division I and division II, and class III. We have not considered open bite, cross-bite, or crowding in this study. The third limitation includes types of sounds: we have considered only labiodental, linguodental, lingua-alveolar, and bilabial sounds; here, guttural sounds like 'G' and 'K' were not considered. The data we collected also show that there is a negative impact of class III malocclusion on speech pronunciation. According to the results, lingua-alveolar sounds are statistically significant and the speech defects in lingua-alveolar sounds are most commonly observed in Angle's class III malocclusion study participants. In Angle's class III, we can find maxillary deficiency and mandibular prognathism. The results of this study are comparable with the other studies in which the subjects in class III malocclusion have difficulty in pronouncing lingua-alveolar sounds like ‘s’ and ‘t’, which is due to anterior constriction or maxillary deficiency, or in some cases, it is also due to over-protrusion of the tongue tip [15]. In addition to this, the subjects with class II malocclusion with midline diastema are shown to ‘lisp’ while producing consonant ‘s’, which is because of the air that escapes through the space present between incisors in the midline diastema. Although the linguodental sounds are not statistically significant, class III malocclusion subjects also show a higher incidence of linguodental speech defects. Most of the subjects have lingua-alveolar speech defects in the production of ‘s’ and ‘t’ sounds.

## Conclusions

Most subjects with class III malocclusion were observed to have the highest incidence of speech defects. Most study participants showed speech difficulties in lingua-alveolar sounds while pronouncing ‘s’ and ‘t’ sounds. Speech defects were commonly affected by different traits of malocclusion, which also affects the quality of life of people. The more severe the malocclusion, the more severe will be the speech defect. Class III malocclusion is the most severe form in which the speech defect occurs due to maxillary deficiency and the protrusion of the tongue.
